# Testing Predictive Factors of Depressive Symptoms among Grandparents under the COVID-19 Pandemic

**DOI:** 10.1093/geroni/igab046.2938

**Published:** 2021-12-17

**Authors:** Yuqin Jiao, Christine Fruhauf

**Affiliations:** Colorado State University, Fort Collins, Colorado, United States

## Abstract

An estimated 69.5 million Americans are reported to be grandparents. Among them, about 10% are raising grandchildren and the number of grandparents who are raising grandchildren (GRG) is increasing. Previous research on GRG suggests that the unexpected caregiving duties may lead to negative physical and mental health including more depressive symptoms when compared to non-caregiving grandparents (NGRG). Additionally, grandparent-grandchild relationships determined by emotional availability (EA) of the grandparent may be impacted. These factors might further be complicated, especially as it relates to the health and well-being of GRG, as a result of the COVID-19 pandemic. Thus, the overarching goal of this presentation is to use the biopsychosocial model to present a conceptual framework to test the mental well-being of GRG during the COVID-19 pandemic. In this presentation, we will 1) summarize appropriate literature on GRG; 2) share a COVID-19 health and well-being assessment survey designated for GRG in order to assess their health before and since the COVID pandemic; and 3) propose a conceptual model to investigate and test the protective role of physical activity and GRG’s EA in the grandparent-grandchild relationship for the mental health of GRG. In our model, we argue that GRG experience more COVID-19 pandemic-related stress and more depressive symptoms when compared to NGRG. This proposed conceptual model offers one way to test the predictors of depressive symptoms on GRG. Future testing has the potential to shed new light on the development of appropriate intervention programs tailored to maintain the mental health of GRG.

